# New Consensus pattern in Spike CoV-2: potential implications in coagulation process and cell–cell fusion

**DOI:** 10.1038/s41420-020-00372-1

**Published:** 2020-11-27

**Authors:** Silvia Buonvino, Sonia Melino

**Affiliations:** Department of Chemical Science and Technologies, University of Rome “Tor Vergata”, via della Ricerca Scientifica 1, 00133 Rome, Italy

**Keywords:** Viral proteins, Infectious diseases

## Abstract

Coagulopathy and syncytial formation are relevant effects of the SARS-CoV-2 infection, but the underlying molecular mechanisms triggering these processes are not fully elucidated. Here, we identified a potential consensus pattern in the Spike S glycoprotein present within the cytoplasmic domain; this consensus pattern was detected in only 79 out of 561,000 proteins (UniProt bank). Interestingly, the pattern was present in both human and bat the coronaviruses S proteins, in many proteins involved in coagulation process, cell–cell interaction, protein aggregation and regulation of cell fate, such as von Willebrand factor, coagulation factor X, fibronectin and Notch, characterized by the presence of the cysteine-rich EGF-like domain. This finding may suggest functional similarities between the matched proteins and the CoV-2 S protein, implying a new possible involvement of the S protein in the molecular mechanism that leads to the coagulopathy and cell fusion in COVID-19 disease.

The severe acute respiratory syndrome coronavirus (SARS-1) of 2002, the Middle East respiratory syndrome coronavirus (MERS-CoV) of 2012, and now the SARS-2 of 2019 (causing the COVID-19 disease) are all due to coronaviruses of the beta subgroup^1^. This positive-sense single-stranded RNA virus family possesses the structural proteins spike (S), membrane (M) and envelope (E) proteins, along with the nucleocapsid (N) protein^2^.

SARS spike glycoprotein is a trimeric protein that shows a large mass of 500 kDa for trimer and, striking, this appears like a three-bladed propeller with a radius of 90 Å^3^ (see Fig. 1). The SARS spike protein is characterized by the presence of four structural domains (Fig. 1). While the two large ecto-domains S1 and S2 are responsible for receptor binding and membrane fusion respectively, the cytoplasmic domain (CD) has an important function in the assembly of several enveloped viruses, as described for other viral membrane proteins. In the case of alphaviruses, for instance, the CD of the E2 glycoprotein plays a pivotal role in the interaction with the capsid protein during particle formation^4^^,5^. A critical role for the cytoplasmic tails in this process has been reported for several members including Simian virus 5 (SV5)^6,7^, Sendai virus^8,9^, and measles virus^10^. In Sendai virus the matrix protein was found to interact independently of the cytoplasmic tails of the HN and F glycoproteins^11,12^. In orthomyxovirus influenza A, the cytoplasmic tails of the two glycoproteins, HA and NA, influence budding efficiency as well as particle morphology. Their separate removal caused only limited effects while the lack of both tails resulted in severely impaired formation of deformed particles^13–16^. Interestingly, many viral ectodomain fragments of fusion protein without transmembrane (TM) and ENDO domains fold into a post-fusion states^17,18^, suggesting that membrane-anchoring parts help maintain functional metastable high energy conformations. In the case of HIV-1-gp41, for instance, Lu et al. report that antibodies (IgG) against LLP1–2 and LLP2 (lentivirus lytic peptide α helix 1 and 2) regions inhibited HIV-1 Envelope-mediated cell fusion and bound to the interface between effector and target cells suggesting that LLP1–2, especially the LLP2 region located inside the viral membrane is transiently exposed on the membrane surface during the fusion process^19^.

**Fig. 1:**
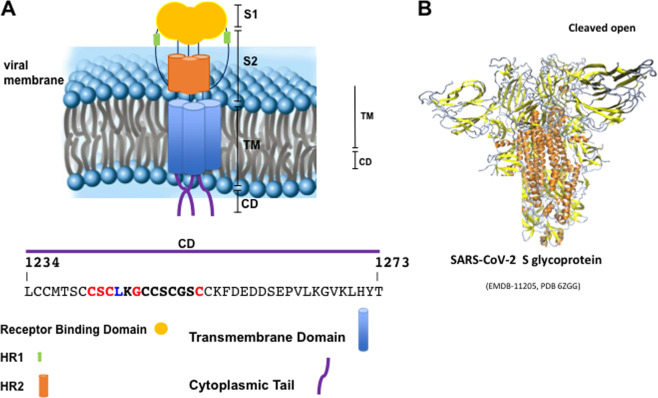
SARS-CoV-2 S protein and cytoplasmic-domain. Schematic representation of the SARS-CoV-2 Spike glycoprotein in the viral membrane (**a**). Cartoon backbone representation of the three-dimensional structure 6ZGG pdb of the S1 and S2 regions in pre-fusion native state in the furin-cleaved open conformation (**b**)^49^. The cytoplasmic tail sequence is reported and the analyzed sequence is highlighted. In red, the consensus pattern is shown. S (subunit), TM (Transmembrane Domain), CD (Cytoplasmic Domain), and HR (heptad repeat region).

For coronaviruses is not entirely clear how the intra-virion parts of the fusion protein influence reactions that are carried out by the much larger exterior portion of the protein.

In the carboxy-terminal domain of the Coronaviral S protein there are two areas of conservation: one is at the transition of TM and ectodomain, i.e. where the S protein exits the viral membrane and it is characterized by a conspicuous, highly conserved 8-residues sequence (KWPWY/WVWL), probably important for membrane fusion but not for S protein incorporation into particles. The other area is located in the membrane-proximal part of the CD and it shows a conserved abundance of cysteines (Fig. 1). The carboxy-terminal truncations reveal that it is this specific domain that mediates particle assembly of the coronaviral spikes.

The importance of the cysteine-rich region for membrane fusion has already been established^20,21^. A spike mutant, with part of the cysteine-rich region deleted, was able to promote hemi-fusion, but was blocked in fusion pore formation. Whether this effect was due by preventing acylation is not completely clear, but it is possible that the membrane-inserted hydrophobic acyl chains are implicated in fusion pore formation. A positive role of cysteine palmitoylation in cell fusion has been reported for influenza virus HA protein^22,23^, while a negative role was observed for Vesicular Stomatitis virus (VSV)^24^, influenza virus^25,26^ and the Murine leukemia virus fusion protein^27^. In CoV S protein, the cysteines, and/or their palmitate adducts of the endo-domain, can change the rate-limiting step of the membrane fusion reaction^28^. Therefore, in this contest the role of the CD should be better investigated.

We analyzed the CD sequence of CoV-2 S protein in order to identify potential consensus sequence patterns (Fig. 1). A bioinformatic analysis using PattInProt v5.4up (https://npsa-prabi.ibcp.fr/cgi-bin/npsa_automat.pl?page=npsa_pattinprot.html)^29^, setting 100% of similarity, was performed identifying, in 79 proteins out of 561,000 proteins of the UniProt bank, a new potential amino acid pattern. The consensus pattern was C-[TS]-C-h-X-G-X(4,6)-C, herein called CAF-motif (Cysteine Aggregation Fusion), where h is a hydrophobic residue and X any other residue. The consensus pattern and the sequence alignments between the proteins and the pattern are shown in Fig. 2 and the PattInProt analysis is shown in Fig. 1S.

**Fig. 2:**
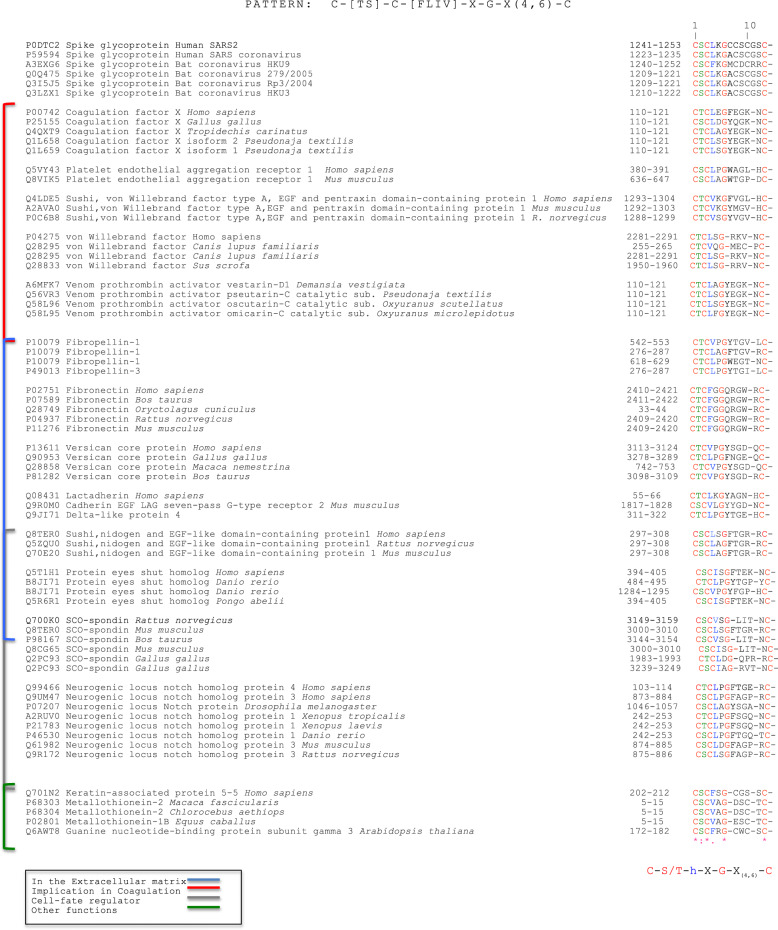
Selected Protein Sequences with their UniProt code that show the CAF-motif. The analysis was performed with PattInProt program^29^. In blue square brackets are indicated proteins present in Extracellular matrix, in red ones those implicated in coagulation, in gray ones regulators of cell fate and in green ones those involved in other biological processes.

Interestingly, the only viral proteins that showed the CAF-motif were S proteins of human coronaviruses SARS and SARS -2 and the S proteins of bat coronavirus. Moreover, other proteins involved in coagulation, extracellular recognition and cell fate presented the same pattern. Intriguingly, the CAF-motif occurs in proteins such as coagulation factor X, von Willebrand factor, platelet endothelial aggregation receptor 1 and some pro-thrombin activators venom toxins that are involved in the coagulation process. The identification of a common pattern could suggest a new function of the S protein in the pathological effects of the SARS -2 infection.

Biopsy and autopsy studies on COVID-19 patients showed pulmonary pathology alveolar damage with diffuse thickening of the alveolar wall, formation of hyaline membranes and macrophages and mononuclear cells infiltration^30–36^. Moreover, according to recent reports, the most severely ill patients show relevant coagulopathy^37,38^. Clinical studies have revealed that 71.4% of non-survivors of COVID-19 matched the grade of overt disseminated intravascular coagulation (≥5 points according to the International Society on Thrombosis and Haemostasis criteria) and showed abnormal coagulation results during later stages of the disease such as particularly increased levels of D-dimer and other fibrin degradation products that were significantly associated with poor prognosis^39,40^. The molecular mechanisms at the base of coagulopathy in COVID-19 disease are not yet identified so that the identification of this common pattern could suggest a similar molecular mechanism in the coagulation induction.

Other matched proteins are found in the extracellular matrix (ECM) involved in cellular adhesion to the matrix, cell–cell interaction, and cell signaling such as: fibropellin, fibronectin, versican, cadherins, and proteins responsible for cell fate regulation such as spondin, nidogen, and the cell-surface receptor Notch, which is also involved in fusion cell fate^41^ and its ligand Dll4 (Delta-like 4).

Among the protein list are metallothioneins and Keratin-associated protein 5-5 (KRTAP 5-5); the first ones are a family of small, highly conserved, cysteine-rich metal-binding proteins important for zinc and copper homeostasis, buffering against toxic heavy metals and protection from oxidative stress, while KRTAP 5-5 belongs to a large protein family involved in crosslinking keratin intermediate filaments during hair formation process.

Intriguingly, this consensus pattern is located in the epidermal growth factor (EGF)-like domain present in the great part of the found proteins and characterized by six cysteines which form disulfide bonds within the domain (C1–C3, C2–C4, and C5–C6).

The EGF-like domain is involved in receptor–ligand interactions, extracellular matrix formation, cell adhesion and signal transduction, and chemotaxis^42^ and in many proteins such as coagulation factors the EGF-like domains are known to bind calcium ions with D and E residues allowing Ca^2+^ mediated protein–protein interactions^43,44^. In Notch, for example, the EGF motifs show multiple functions, such as the prevention of constitutive activation, reciprocal interaction with the ligands, and lateral interaction for homodimerization, playing a crucial role in Notch signaling system^45^. Moreover, Notch signaling, a major regulator of cardiovascular function and inflammation, is also implicated in several biological processes mediating viral infections such as SARS-CoV-2, playing an important role in developing of myocarditis, heart failure, and lung inflammation in COVID-19 patients^46^. In macrophages Dll1,4/Notch signaling promotes the inflammatory cytokines storm, interleukin-6 (IL-6) among those, which in turn increases the expression of notch ligands (Dll1,4), thus amplifying the signal establishing a feedback loop^46^.

In view of the above, we propose a hypothetic active role of the Coronavirus S protein cytoplasmic domain in protein–protein aggregation for clots formation and cell–cell fusion SARS-2-S protein-driven^47^. Therefore, our findings suggest a new potential molecular mechanism linked to the infection in which after virus-cell fusion, the infected cells expose on their surface the CAF-motif, leading to clots formation and cell–cell fusion by protein–protein aggregation processes. Moreover, it should not to be rolled out the ability of CD’s S protein to coordinate Ca^2+^ ions, similarly to EGF-like domain, for mediating cell–cell fusion, which is responsible for instance of syncytia formation^48^.

The identification of this new consensus pattern provides a first evidence of a functional similarity between the CoV S protein and proteins involved in coagulation, in regulation of cell fate and cell–cell interaction and fusion that are at the base of coagulopathy and syncytia formation in COVID-19 disease.

## Supplementary information

Figure 1S. The original PattInProt analysis
