# Supplementary material for: New Consensus pattern in Spike CoV-2: potential implications in coagulation process and cell–cell fusion
Source: Cell Death Discov. 2020 Nov 27;6:134. doi: 10.1038/s41420-020-00372-1 (PMC7691694; doi:10.1038/s41420-020-00372-1)
Supplement: Supplementary file 1 — Figure 1S. The original PattInProt analysis [file 41420_2020_372_MOESM1_ESM.pdf]

```
ID   CAF-; PATTERN DE   NONE  PA   C-[TS]-C-[FLIV]-X-G-X(4,6)-C  CC
 Randomized probability: min = 5.551e-09 max = 5.560e-09 . CC   Patte
rns In proteins query (PattInProt v5.4up, November 15th, 2011) CC   C
ontact Dr. Christophe Blanchet, christophe.blanchet@ibcp.fr, http://g
bio-pbil.ibcp.fr/cblanchet CC   Scan criterion: Similarity 100% CC
Sequence databank: /db/uniprot/fasta/current/swissprot.fas CC   Numbe
r of sequences in databank: 561356 (Tue, 29 Sep 2020 10:07:29 AM UTC)
.                                                 //
```

First step : before [go to EXTRACT].

SELECT SITES of pattern 1 with 100% similarity.

```
---------------------------------------------------------------------
----------- NPSA gnl|unipsp|P40410  [Bacteria] Iron-uptake system per
mease protein FeuB [Firmicutes][Bacillus subtilis] (length=334 residu
es).  *****> PATTERN 1    Site :   282-  294, Identity    vgsdysrli
p_CSCILGGIFLTLC_dlasrfinyp ------------------------------------------
-------------------------------------- NPSA gnl|unipsp|P47104  [Eukar
yota] Regulator of V-ATPase in vacuolar membrane protein 1 [Fungi][Sa
ccharomyces cerevisiae] (length=1357 residues).  *****> PATTERN 1
  Site :   602-  614, Identity    aceintgimn_CTCIRGSSTGKLC_ivnstgkvms
 --------------------------------------------------------------------
------------ NPSA gnl|unipsp|A2VEC9  [Eukaryota] SCO-spondin [Metazoa
][Homo sapiens] (length=5150 residues).  *****> PATTERN 1    Site :
  3165- 3175, Identity    gstvvrpcen_CSCVSGLIANC_sswpcaegep ---------
---------------------------------------------------------------------
-- NPSA gnl|unipsp|P00742  [Eukaryota] Coagulation factor X [Metazoa]
[Homo sapiens] (length=488 residues).  *****> PATTERN 1    Site :
 110-  121, Identity    kckdglgeyt_CTCLEGFEGKNC_elftrklcsl ----------
---------------------------------------------------------------------
- NPSA gnl|unipsp|P02751  [Eukaryota] Fibronectin [Metazoa][Homo sapi
ens] (length=2477 residues).  *****> PATTERN 1    Site :  2410- 242
1, Identity    qkeylgaics_CTCFGGQRGWRC_dncrrpggep -------------------
------------------------------------------------------------- NPSA gn
l|unipsp|P04275  [Eukaryota] von Willebrand factor [Metazoa][Homo sap
iens] (length=2813 residues).  *****> PATTERN 1    Site :  2281- 22
91, Identity    wvpdhqpcqi_CTCLSGRKVNC_ttqpcptaka -------------------
------------------------------------------------------------- NPSA gn
l|unipsp|P13611  [Eukaryota] Versican core protein [Metazoa][Homo sap
iens] (length=3396 residues).  *****> PATTERN 1    Site :  3113- 31
24, Identity    tcyptetsyv_CTCVPGYSGDQC_eldfdechsn ------------------
-------------------------------------------------------------- NPSA g
nl|unipsp|Q08431  [Eukaryota] Lactadherin [Metazoa][Homo sapiens] (le
```

ngth=387 residues). *****> PATTERN 1 Site : 55- 66, Identi
ty vrgdvfpsyt_CTCLKGYAGNHC_etkcveplgl ---------------------------
--- NPSA gnl|unipsp|
Q2I0M4 [Eukaryota] Leucine-rich repeat-containing protein 26 [Metazo
a][Homo sapiens] (length=334 residues). *****> PATTERN 1 Site :
47- 57, Identity slgapdcpev_CTCVPGGLASC_salslpavpp ---------

-- NPSA gnl|unipsp|Q4LDE5 [Eukaryota] Sushi, von Willebrand factor t
ype A, EGF and pentraxin domain-containing protein 1 [Metazoa][Homo s
apiens] (length=3571 residues). *****> PATTERN 1 Site : 1293-
1304, Identity icvdgvagyr_CTCVKGFVGLHC_etevnecqsn ----------------
--- NPSA
gnl|unipsp|Q5IJ48 [Eukaryota] Protein crumbs homolog 2 [Metazoa][Ho
mo sapiens] (length=1285 residues). *****> PATTERN 1 Site : 11
59- 1170, Identity pceggspaan_CSCLEGLAGQRC_qvptlpcean ------------
----
NPSA gnl|unipsp|Q5T1H1 [Eukaryota] Protein eyes shut homolog [Metazo
a][Homo sapiens] (length=3165 residues). *****> PATTERN 1 Site
: 394- 405, Identity tckkcekdyp_CSCISGFTEKNC_ekaidhckll -------
----
---- NPSA gnl|unipsp|Q5VY43 [Eukaryota] Platelet endothelial aggrega
tion receptor 1 [Metazoa][Homo sapiens] (length=1037 residues). ****
*> PATTERN 1 Site : 380- 391, Identity slschpmnge_CSCLPGWA
GLHC_nescpqdthg ---
--------------------------- NPSA gnl|unipsp|Q701N2 [Eukaryota] Kerat
in-associated protein 5-5 [Metazoa][Homo sapiens] (length=237 residue
s). *****> PATTERN 1 Site : 202- 212, Identity yccqssccckp
_CSCFSGCGSSC_cqsscykpcc Site : 202- 213, Identity yccqsscc
kp_CSCFSGCGSSCC_qsscykpccc ---
-------------------------------------- NPSA gnl|unipsp|Q8TER0 [Eukar
yota] Sushi, nidogen and EGF-like domain-containing protein 1 [Metazo
a][Homo sapiens] (length=1413 residues). *****> PATTERN 1 Site
: 297- 308, Identity dcvtgnpsyt_CSCLSGFTGRRC_hldvnecasq -------
----
---- NPSA gnl|unipsp|Q99466 [Eukaryota] Neurogenic locus notch homol
og protein 4 [Metazoa][Homo sapiens] (length=2003 residues). *****>
PATTERN 1 Site : 103- 114, Identity spspltpsfl_CTCLPGFTGER
C_qakledpcpp --
------------------------ NPSA gnl|unipsp|Q9UM47 [Eukaryota] Neurogen
ic locus notch homolog protein 3 [Metazoa][Homo sapiens] (length=2321
residues). *****> PATTERN 1 Site : 873- 884, Identity sc
qdgvgsfs_CSCLPGFAGPRC_ardvdeclsn ------------------------------------
-- NPSA gnl|unipsp|P07207
[Eukaryota] Neurogenic locus Notch protein [Metazoa][Drosophila melan
ogaster] (length=2703 residues). *****> PATTERN 1 Site : 1046-
1057, Identity scidgingyn_CSCLAGYSGANC_qyklnkcdsn ----------------
--- NPS

A gnl|unipsp|P10040 [Eukaryota] Protein crumbs [Metazoa][Drosophila melanogaster] (length=2146 residues). *****> PATTERN 1 Site : 1900- 1911, Identity fnastgnnft_CTCVPGFEGPLC_dipfceitpc --- NPSA gnl|unipsp|P10079 [Eukaryota] Fibropellin-1 [Metazoa][Strongylocentrotus purpuratus] (length=1064 residues). *****> PATTERN 1 Site : 276- 287, Identity icvdgvnmfe_CTCLAGFTGVRC_evnidecasa Site : 542- 553, Identity tctdqvngyv_CTCVPGYTGVLC_etdinecasf Site : 618- 629, Identity acmdvvngfv_CTCLPGWEGTNC_eintdecass -- NPSA gnl|unipsp|P49013 [Eukaryota] Fibropellin-3 [Metazoa][Strongylocentrotus purpuratus] (length=570 residues). *****> PATTERN 1 Site : 276- 287, Identity icvdginqfa_CTCLPGYTGILC_etdinecass -- NPSA gnl|unipsp|P84031 [Eukaryota] U7-ctenitoxin-Pr1a [Metazoa][Phoneutria reidyi] (length=58 residues). *****> PATTERN 1 Site : 38- 48, Identity wfggkwhplg_CSCVYGDKYIC_ekkkkecpnv -- NPSA gnl|unipsp|Q5Y4U3 [Eukaryota] U9-agatoxin-Ao1a [Metazoa][Agelena orientalis] (length=110 residues). *****> PATTERN 1 Site : 70- 80, Identity cscplfgalg_CSCIIGDAMVC_vrkkkecrts -- NPSA gnl|unipsp|Q6TMJ6 [Eukaryota] SrfA-induced gene K protein [Amoebozoa][Dictyostelium discoideum] (length=426 residues). *****> PATTERN 1 Site : 388- 399, Identity sspfiicngt_CTCLPGFSGNDC_tlcgnggevi -- NPSA gnl|unipsp|O19045 [Eukaryota] Coagulation factor X [Metazoa][Oryctolagus cuniculus] (length=490 residues). *****> PATTERN 1 Site : 110- 121, Identity tckdglgmyt_CSCVEGYEGQDC_epvtrklcsl -- NPSA gnl|unipsp|P02801 [Eukaryota] Metallothionein-1B [Metazoa][Equus caballus] (length=61 residues). *****> PATTERN 1 Site : 5- 15, Identity <mdpn_CSCVAGESCTC_agsckckqcr -- NPSA gnl|unipsp|P07589 [Eukaryota] Fibronectin [Metazoa][Bos taurus] (length=2478 residues). *****> PATTERN 1 Site : 2411- 2422, Identity qkeylgaics_CTCFGGQRGWRC_dncrrpgaep -- NPSA gnl|unipsp|P68303 [Eukaryota] Metallothionein-2 [Metazoa][Macaca fascicularis] (length=61 residues). *****> PATTERN 1 Site : 5- 15, Identity <mdpn_CSCVAGDSCTC_agsckckeck -- NPSA gnl|unipsp|P68304 [Eukaryota] Metallothionein-2 [Metazoa][Chlorocebus aethiops] (length=61 residues). *****> PATTERN 1 Site : 5- 15, Identity <mdpn_CSCVAGDSCTC_agsckckeck ---

---------------------------- NPSA gnl|unipsp|P81282 [Eukaryota] Vers
ican core protein [Metazoa][Bos taurus] (length=3381 residues). ****
*> PATTERN 1   Site : 3098- 3109, Identity   tcyptetsyv_CTCVPGYS
GDRC_eldfdechsn ---
--------------------------- NPSA gnl|unipsp|P98167 [Eukaryota] SCO-s
pondin [Metazoa][Bos taurus] (length=5146 residues). *****> PATTERN
1   Site : 3144- 3154, Identity   gsavlrpcen_CSCVSGLITNC_tswpcke
gqp ---
--------------- NPSA gnl|unipsp|Q28295 [Eukaryota] von Willebrand fa
ctor [Metazoa][Canis lupus familiaris] (length=2813 residues). *****
> PATTERN 1   Site :   255- 265, Identity   pfvalcertl_CTCVQGMEC
PC_avlleyarac   Site : 2281- 2291, Identity   wvpahqpcqi_CTCLSGR
KVNC_tlqpcptara ---
--------------------------- NPSA gnl|unipsp|Q28749 [Eukaryota] Fibro
nectin [Metazoa][Oryctolagus cuniculus] (length=100 residues). *****
> PATTERN 1   Site :   33-   44, Identity   qkeylgaics_CTCFGGQRG
WRC_dncrrpgvep --
-------------------------- NPSA gnl|unipsp|Q28833 [Eukaryota] von Wi
llebrand factor [Metazoa][Sus scrofa] (length=2482 residues). *****>
 PATTERN 1   Site : 1950- 1960, Identity   wvpdhqpcqi_CTCLSGRRVN
C_tlqpcptara --
------------------------ NPSA gnl|unipsp|Q28858 [Eukaryota] Versican
 core protein [Metazoa][Macaca nemestrina] (length=862 residues). **
***> PATTERN 1   Site :   742- 753, Identity   tcyptetsyv_CTCVPG
YSGDQC_eldfdechsn ---
------------------------------ NPSA gnl|unipsp|Q5R6R1 [Eukaryota] Pro
tein eyes shut homolog [Metazoa][Pongo abelii] (length=3164 residues)
. *****> PATTERN 1   Site :   394- 405, Identity   tckkwekdyh_C
SCISGFTEKNC_ekaidhcrll --
----------------------------------- NPSA gnl|unipsp|Q6AWT8 [Eukaryota
] Guanine nucleotide-binding protein subunit gamma 3 [Viridiplantae][
Arabidopsis thaliana] (length=251 residues). *****> PATTERN 1   S
ite :   172- 182, Identity   cccprlscps_CSCFRGCWCSC_pdmsccipsc ----
----
------- NPSA gnl|unipsp|Q6NPN4 [Eukaryota] LysM domain-containing GP
I-anchored protein 3 [Viridiplantae][Arabidopsis thaliana] (length=42
3 residues). *****> PATTERN 1   Site :   251- 262, Identity   y
altaghcvq_CSCVLGSRSMYC_epasisvscs -----------------------------------
-- NPSA gnl|unipsp|A2AVA0
 [Eukaryota] Sushi, von Willebrand factor type A, EGF and pentraxin d
omain-containing protein 1 [Metazoa][Mus musculus] (length=3567 resid
ues). *****> PATTERN 1   Site : 1292- 1303, Identity   tctdglas
yr_CTCVKGYMGVHC_etdvnecqss --
------------------------------------- NPSA gnl|unipsp|D3ZHH1 [Eukar
yota] Delta-like protein 4 [Metazoa][Rattus norvegicus] (length=686 r
esidues). *****> PATTERN 1   Site :   311- 322, Identity   csns
gprgyt_CTCLPGYTGEHC_elelskcasn -------------------------------------

-- NPSA gnl|unipsp|P04937 [E
ukaryota] Fibronectin [Metazoa][Rattus norvegicus] (length=2477 resid
ues). *****> PATTERN 1 Site : 2409- 2420, Identity qkeylgai
cs_CTCFGGQRGWRC_dncrrpgaae --
-------------------------------------- NPSA gnl|unipsp|P0C6B8 [Eukar
yota] Sushi, von Willebrand factor type A, EGF and pentraxin domain-c
ontaining protein 1 [Metazoa][Rattus norvegicus] (length=3564 residue
s). *****> PATTERN 1 Site : 1288- 1299, Identity tctdglasyr
_CTCVSGYVGVHC_etdvnecqss ---
------------------------------------ NPSA gnl|unipsp|P11276 [Eukaryo
ta] Fibronectin [Metazoa][Mus musculus] (length=2477 residues). ****
*> PATTERN 1 Site : 2409- 2420, Identity qkeylgaics_CTCFGGQR
GWRC_dncrrpgaae ---
--------------------------- NPSA gnl|unipsp|Q5ZQU0 [Eukaryota] Sushi
, nidogen and EGF-like domain-containing protein 1 [Metazoa][Rattus n
orvegicus] (length=1403 residues). *****> PATTERN 1 Site : 29
7- 308, Identity dcvtgnpsyt_CSCLAGFTGRRC_hldvnecash -------------
--- N
PSA gnl|unipsp|Q61982 [Eukaryota] Neurogenic locus notch homolog pro
tein 3 [Metazoa][Mus musculus] (length=2318 residues). *****> PATTER
N 1 Site : 874- 885, Identity scqdgvgsfs_CSCLDGFAGPRC_ardv
declss ---
------------------ NPSA gnl|unipsp|Q700K0 [Eukaryota] SCO-spondin [M
etazoa][Rattus norvegicus] (length=5141 residues). *****> PATTERN 1
Site : 3149- 3159, Identity gsavilpcen_CSCVSGLITNC_sswpceegq
p ---
------------- NPSA gnl|unipsp|Q70E20 [Eukaryota] Sushi, nidogen and
EGF-like domain-containing protein 1 [Metazoa][Mus musculus] (length=
1403 residues). *****> PATTERN 1 Site : 297- 308, Identity
dcvtgnpsyt_CSCLAGFTGRRC_hldvnecash ------------------------------
-- NPSA gnl|unipsp|Q8CG
65 [Eukaryota] SCO-spondin [Metazoa][Mus musculus] (length=4998 resi
dues). *****> PATTERN 1 Site : 3000- 3010, Identity gsavhlp
cen_CSCISGLITNC_sswpceegqp --
-------------------------------------- NPSA gnl|unipsp|Q8VIK5 [Eukar
yota] Platelet endothelial aggregation receptor 1 [Metazoa][Mus muscu
lus] (length=1034 residues). *****> PATTERN 1 Site : 636- 64
7, Identity hsschpsdgt_CSCLAGWTGPDC_seacppghwg -------------------
-- NPSA gn
l|unipsp|Q9JI71 [Eukaryota] Delta-like protein 4 [Metazoa][Mus muscu
lus] (length=686 residues). *****> PATTERN 1 Site : 311- 322
, Identity csnsgpkgyt_CTCLPGYTGEHC_elglskcasn -------------------
-- NPSA gnl
|unipsp|Q9R0M0 [Eukaryota] Cadherin EGF LAG seven-pass G-type recept
or 2 [Metazoa][Mus musculus] (length=2919 residues). *****> PATTERN
1 Site : 1817- 1828, Identity ycsndwdsys_CSCVLGYYGDNC_tnvcdl
npce --

---------------- NPSA gnl|unipsp|Q9R172 [Eukaryota] Neurogenic locus
 notch homolog protein 3 [Metazoa][Rattus norvegicus] (length=2319 re
sidues). *****> PATTERN 1   Site :   875- 886, Identity   scqdg
vgsfs_CSCLSGFAGPRC_ardvdeclss ---------------------------------------
--- NPSA gnl|unipsp|A2RUV0 [Eu
karyota] Neurogenic locus notch homolog protein 1 [Metazoa][Xenopus t
ropicalis] (length=2522 residues). *****> PATTERN 1   Site :   24
2- 253, Identity   crqtddtsye_CTCLPGFSGQNC_eeniddcpsn -------------
--- N
PSA gnl|unipsp|A6MFK7 [Eukaryota] Venom prothrombin activator vestar
in-D1 [Metazoa][Demansia vestigiata] (length=473 residues). *****> P
ATTERN 1   Site :   110- 121, Identity   tckdgigsyt_CTCLAGYEGKNC
_ehdllkscrv ---
----------------------- NPSA gnl|unipsp|A6MFK8 [Eukaryota] Venom pro
thrombin activator vestarin-D2 [Metazoa][Demansia vestigiata] (length
=471 residues). *****> PATTERN 1   Site :   110- 121, Identity
  tckdgigsyt_CTCLPGYEGKNC_ehvvvkscrl --------------------------------
-- NPSA gnl|unipsp|B8JI
71 [Eukaryota] Protein eyes shut homolog [Metazoa][Danio rerio] (len
gth=2904 residues). *****> PATTERN 1   Site :   484- 495, Identi
ty   sqttapphym_CTCLPGYTGPYC_eaevnecdss   Site : 1284- 1295, Ide
ntity   vcqdlvngfq_CSCVPGYFGPHC_nldvnecdss -------------------------
--- NPSA gnl|unip
sp|P21783 [Eukaryota] Neurogenic locus notch homolog protein 1 [Meta
zoa][Xenopus laevis] (length=2524 residues). *****> PATTERN 1   S
ite :   242- 253, Identity   crqtddtsyd_CTCLPGFSGQNC_eeniddcpsn ---
----
-------- NPSA gnl|unipsp|P25155 [Eukaryota] Coagulation factor X [Me
tazoa][Gallus gallus] (length=475 residues). *****> PATTERN 1   S
ite :   110- 121, Identity   qckdglgsyt_CSCLDGYQGKNC_efvipkycki ---
----
-------- NPSA gnl|unipsp|P46530 [Eukaryota] Neurogenic locus notch h
omolog protein 1 [Metazoa][Danio rerio] (length=2437 residues). ****
*> PATTERN 1   Site :   242- 253, Identity   cvqtsdttht_CSCLPGFT
GQTC_ehnvddctqh ---
--------------------------- NPSA gnl|unipsp|Q1L658 [Eukaryota] Coagu
lation factor X isoform 2 [Metazoa][Pseudonaja textilis] (length=463
residues). *****> PATTERN 1   Site :   110- 121, Identity   ick
dgigsyt_CTCLSGYEGKNC_ervlykscrv -------------------------------------
--- NPSA gnl|unipsp|Q1L659 [
Eukaryota] Coagulation factor X isoform 1 [Metazoa][Pseudonaja textil
is] (length=483 residues). *****> PATTERN 1   Site :   110- 121,
 Identity   tckdgigsyt_CTCLSGYEGKNC_eyvlykscrv ---------------------
--- NPSA gnl|
unipsp|Q2PC93 [Eukaryota] SCO-spondin [Metazoa][Gallus gallus] (leng
th=5255 residues). *****> PATTERN 1   Site : 1983- 1993, Identit

y gqrmklncrl_CTCLDGQPRRC_rhnpacsvsc Site : 3239- 3249, Ident
ity gdvvpqgchn_CSCIAGRVTNC_sqedcgdvdg --------------------------
-- NPSA gnl|unipsp|
Q4QXT9 [Eukaryota] Coagulation factor X [Metazoa][Tropidechis carina
tus] (length=483 residues). *****> PATTERN 1 Site : 110- 121
, Identity tckdgigsyt_CTCLAGYEGKNC_qyvlyqscrv --------------------
-- NPSA gnl
|unipsp|Q56VR3 [Eukaryota] Venom prothrombin activator pseutarin-C c
atalytic subunit [Metazoa][Pseudonaja textilis] (length=467 residues)
. *****> PATTERN 1 Site : 110- 121, Identity ickdgigsyt_C
TCLSGYEGKNC_ervlykscrv --
---------------------------------- NPSA gnl|unipsp|Q58L95 [Eukaryota
] Venom prothrombin activator omicarin-C catalytic subunit [Metazoa][
Oxyuranus microlepidotus] (length=467 residues). *****> PATTERN 1
Site : 110- 121, Identity tckdgigsyt_CTCLFGYEGKNC_ervlyksc
rv --
-------------- NPSA gnl|unipsp|Q58L96 [Eukaryota] Venom prothrombin
activator oscutarin-C catalytic subunit [Metazoa][Oxyuranus scutellat
us] (length=467 residues). *****> PATTERN 1 Site : 110- 121,
Identity tckdgigsyt_CTCLSGYEGKNC_ervlykscrv ---------------------
--- NPSA gnl|
unipsp|Q90953 [Eukaryota] Versican core protein [Metazoa][Gallus gal
lus] (length=3562 residues). *****> PATTERN 1 Site : 3278- 328
9, Identity tcyprgsfyi_CTCLPGFNGEQC_eldidecqsn -------------------
-- NPSA gn
l|unipsp|A3EXG6 [Viruses] Spike glycoprotein [Riboviria][Bat coronav
irus HKU9] (length=1274 residues). *****> PATTERN 1 Site : 124
0- 1252, Identity vimlmcmtnc_CSCFKGMCDCRRC_cgsydsyddv ------------
----
NPSA gnl|unipsp|P59594 [Viruses] Spike glycoprotein [Riboviria][Huma
n SARS coronavirus] (length=1255 residues). *****> PATTERN 1 Si
te : 1223- 1235, Identity tillccmtsc_CSCLKGACSCGSC_ckfdeddsep ---
----
-------- NPSA gnl|unipsp|Q0Q475 [Viruses] Spike glycoprotein [Ribovi
ria][Bat coronavirus 279/2005] (length=1241 residues). *****> PATTER
N 1 Site : 1209- 1221, Identity tillccmtsc_CSCLKGACSCGSC_ckf
deddsep --
------------------- NPSA gnl|unipsp|Q3I5J5 [Viruses] Spike glycoprot
ein [Riboviria][Bat coronavirus Rp3/2004] (length=1241 residues). **
***> PATTERN 1 Site : 1209- 1221, Identity tillccmtsc_CSCLKG
ACSCGSC_ckfdeddsep ---
------------------------------ NPSA gnl|unipsp|Q3LZX1 [Viruses] Spik
e glycoprotein [Riboviria][Bat coronavirus HKU3] (length=1242 residue
s). *****> PATTERN 1 Site : 1210- 1222, Identity tillccmtsc
_CSCLKGACSCGSC_ckfdeddsep ---
------------------------------------ Total number of sequences in PA

```
TTINPROT run 1 : 72 Total number of patterns found in PATTINPROT run
1 : 78 Total number of selected patterns for pattern 1 : 78
```

Figure 1S. The original PattInProt analysis using the pattern C-[TS]-C-[FLIV]-X-G-X(4,6)-C.
